# Reorganising health and social care in Québec: a journey towards integrating care through mergers

**DOI:** 10.1080/17571472.2018.1453957

**Published:** 2018-03-23

**Authors:** Paul Wankah, Maxime Guillette, Sophie Dumas, Yves Couturier, Dominique Gagnon, Louise Belzile, Yahya Mosbah, Mylaine Breton

**Affiliations:** 1 Centre de recherche Charles-Le Moyne – Saguenay–Lac-Saint-Jean sur les innovations en santé, Université de Sherbrooke, Longueuil, Canada; 2 Centre de Recherche sur le vieillissement du CIUSS-CHUS de l’Estrie, Université de Sherbrooke, Sherbrooke, Canada; 3 Université de Québec en Abitibi-Témiscamingue, Abitibi, Canada; 4 Université de Sherbrooke, Sherbrooke, Canada

**Keywords:** Integrated care, health system reforms, centralised governance

## Abstract

**Context:**

Two reforms (2014, 2015) characterised by the merger of public health care establishments profoundly shaped the current organisation of Quebec’s healthcare system. In 2015, 22 megastructures called Integrated Health and Social Services Centres/Integrated University Health and Social Services Centres (IHSSC/IUHSSC), were created and mandated to organise care delivery to their local populations.

**Objective:**

To describe the service configuration of the 2015 healthcare system reforms, emphasising on how it shaped the organisation of primary health care (PHC) in Quebec.

**Results:**

With the creation of IHSSCs/IUHSSCs, Quebec’s healthcare system passed from three to two levels of governance, leading to a centralisation of decision-making powers. Most health services are delivered by the new organisations, while most PHC is delivered by semi-private medical practices, mainly Family Medicine Groups (FMGs). The FMG model is the preferred strategy to develop interdisciplinary team-work and inter-organizational collaborations with other PHC services.

**Conclusion:**

mechanisms through which centralised healthcare systems achieve community oriented integrated care (COIC) need to be properly understood in order to improve meaningful clinical outcomes. Mergers may not sufficiently achieve integration of services in all its dimensions. These reforms should be monitored and evaluated on their capacity to mobilise all providers as well as physicians to participate in COIC.

**Why this matters to me**

Strengthening Primary Health Care systems through Community Oriented Integrated Care (COIC) is a promising avenue to address the health needs of ageing population. It is important to understand the mechanisms and strategies underlying the adoption of COIC models, so as to improve their translation to effective clinical outcomes. This article describes the 2015 Quebec healthcare system structural reforms, merging all public health care organisations of a regional territory under a single governance, and strategies to enhance the participation of primary care physicians in COIC. How do healthcare organisations address their local specificities in a context of centralised governance? How do local communities participate in centralised healthcare systems? How can we enhance the participation of primary care physicians in COIC? We think monitoring and evaluation of healthcare systems will aid in improving our understanding of how centralised healthcare systems may impact meaningful integration in local communities.

**Key message**

Healthcare system approaches to improve community oriented integrated care through organisational mergers and strategies to enhance the participation of primary health care providers should be monitored and evaluated, so as to improve meaningful integration in local communities.

## Introduction

Developed countries are constantly reorganising their healthcare systems. In line with the *quadruple aims of healthcare reform goals*, these countries implicitly or explicitly aim to improve patient care experiences, population health, the healthcare system cost efficiency, and the working lives of health care providers [1]. This usually involves the implementation, routinization, and sustainability of innovative organisational care delivery models. Community Oriented Integrated Care (COIC) is an organisational care delivery model that promotes inter-professional and inter-organisational collaborations, coordination and interdependence in delivering a comprehensive package of services to a specified population in a given territory [2,3]. In other words, ‘care that is integrated at the community level’ [4, p.55].

Several health system reforms aimed at achieving the *quadruple aims of healthcare reform goals* have been implemented in Canadian provinces. In a previous LJPC paper, Breton et al. [5] described the large-scale 2004 healthcare system reforms in the province of Quebec that resulted in the creation of 94 Health and Social Service Centres (HSSC) through the merger of local community health centres, long-term care facilities and some acute care hospitals. The paper also described the introduction of new primary care models promoting family physician group practices such as Family Medicine Groups (FMGs) and network clinics, and integrated Local Health Networks (LHNs) to promote the creation of local strategic alliances between HSSCs, FMGs, and community organisations. This was intended to improve the delivery of integrated health and social care at the community level through inter-organisational collaborations with primary health care (PHC) organisations [6].

In 2015, another healthcare system reform was implemented in Quebec. This paper describes the impact of this reform on the current organisation of COIC in the province, in order to stimulate discussion about conditions that help COIC to thrive.

## Context

With a population of about 8.3 million people [7], Quebec is the second most populous province in Canada. Most healthcare services in Quebec are funded through a Beveridgean public health insurance system. Essential care, whether offered at public institutions or private medical clinics, is usually free at the point of service [8]. From its inception in 1971, the modern Quebec healthcare system was designed to integrate healthcare and social services in a primary care structure based on a territorial approach [9]. There are various organisational funding models. For instance, public not-for-profit organisations such as Health and Social Service Centres receive block funding from the government, while private for-profit organisations such as community organisations may receive mixed funding (public and private funds) [10]. There are various complex co-existing models for the remuneration of physicians, consisting mainly in fees-for-service and salaries, with capitation in a few cases. For instance, physicians who work in hospitals and private clinics are mainly remunerated on a fee-for-service basis, paid for by the public health insurance. Some physicians who work in local health and social service centres receive salaries. Furthermore, family physicians earn bonuses for specified activities such as enrolling new patients into their panels [11]. On the other hand, other providers who work in public organisations (e.g. nurses, social workers, occupational therapists) are mainly remunerated through salary.

The socio-political context of Québec has been marked by the implementation of successive structural healthcare reforms. In parallel to this, several strategies have been put in place to improve PHC services and enhance COIC. The development of FMG models based on the Patient Centred Medical Homes model was the core strategy [12] to improve PHC from 2002. The complex socio-political context of Quebec’s healthcare system is beyond the scope of this paper. This manuscript focuses on the description of the two major structural reforms of 2004 and 2015, which led to organisational mergers, and two parallel reforms aimed at promoting the participation of family physicians in COIC.

## Major structural reforms of Quebec’s healthcare system

In 2004, the Quebec government initiated a significant reform by setting up integrated LHNs across the province [5,13]. The territories of covered by HSSCs varied from semi-urban areas with low population densities covering tens of thousands of people to mega-urban areas with high population densities covering hundreds of thousands of people [14]. As previously reported, 94 new organisations called Health and Social Services Centres (HSSCs), were created through the merger of territorially-based local health and social services centres, long-term care institutions, and, in 85% of cases, an acute care hospital. Each HSSC was responsible for the organisation of COIC in its territory. They were formally mandated to promote the implementation of LHNs for specific population groups, through the creation of formal or informal inter-professional and inter-organisational alliances in their territories. The participation of physicians in COIC was promoted through strategic partnerships between HSSCs and FMGs/network clinics on their territories. Nonetheless, family physicians were still very much peripheral with respect to COIC initiatives in Quebec.

**Figure 1.**
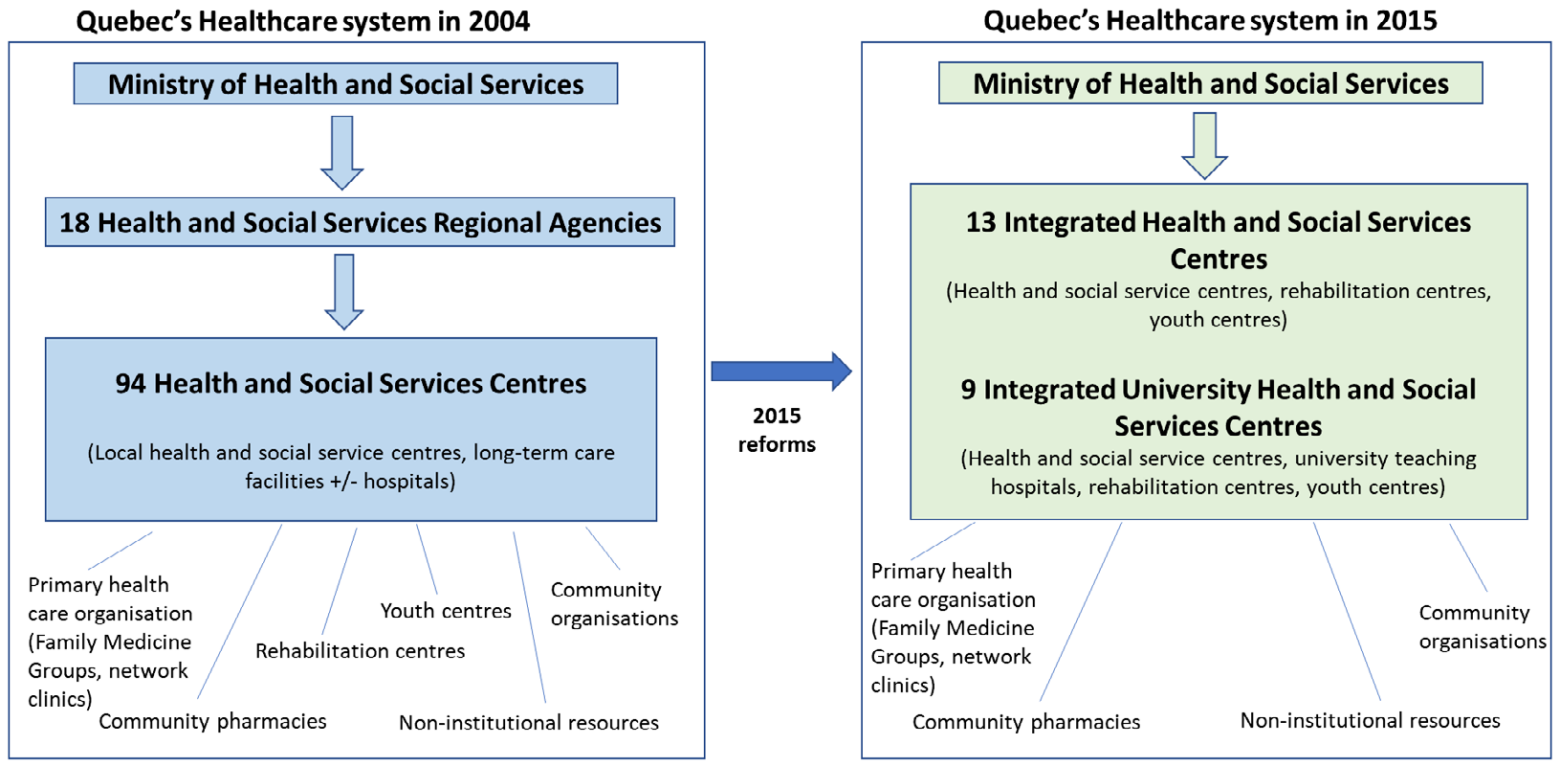
Illustration of the macro organisation of Quebec’s healthcare system in 2004 and 2015.

Quebec’s health and social services system was again deeply reorganised in 2015. The government pursued a centralisation process that abolished Regional Health Authorities. Turgeon et al. reported that there was a major trend towards centralised governance of health systems in all Canadian provinces except Ontario and to a lesser extent British Columbia [10]. In fact, they noticed that ‘the direct authority of the minister (on local healthcare organisations in Quebec) testifies to an unprecedented situation in the last fifty years’ [10, p.194]. Basically, Quebec’s healthcare system passed from three levels of governance (Ministry, regional agencies, and Health and Social Services Centres) to two (Ministry, and Integrated Health and Social Service Centres) (see Figure 1) [10]. This was accomplished by merging neighbouring Health and Social Services Centres, to all the other public healthcare organisations, such as youth centres, rehabilitation centres and university teaching hospitals under a single governing body per regional territory. As a result, the territory of Québec is now divided into 22 functional units with the creation of 13 Integrated Health and Social Service Centres (IHSSCs), and 9 Integrated University Health and Social Service Centres (IUHSSCs, which have additional research and training roles).These new organisations form strategic partnerships with community organisations and private organisations on their territories The government of Quebec explicitly stated that this reorganization was put in place to ‘facilitate and simplify access of services to the population, to improve the quality and security of services, and to increase the efficacy and efficiency of the health system’ [15]. Figure 1 illustrates the structural changes.

## Functional changes

These 22 new health organisations (13 IHSSCs and 9 IUHSSCs) took over the mandates and missions of the previous structures in their areas of jurisdiction. The new organisations gathered a broader scope of public services under a single governing body per territory. Specifically:

• The IHSSCs/IUHSSCs assumed the role of the now-defunct Health and Social Services Regional Agencies in the organisation of human and material resources, and the implementation of health policies according to the specific needs of their local populations. Other roles of the former Health and Social Services Regional Agencies, such as the distribution of financial resources to healthcare organisations on their territories, were taken over by the Ministry of Health and Social Services of Quebec.
• The IHSSCs/IUHSSCs ensure delivery of acute care, community-based care, home care services, rehabilitation services, training of health personnel, as well as health prevention and promotion services through the various establishments (hospitals, local community health and social services centres, long-term care facilities, day hospitals, rehabilitation centres) under their respective governance.
• The IHSSCs/IUHSSCs assumed the role of the former Health and Social Services Centres in the organisation of LHNs for specific vulnerable populations (e.g. Local Health Network for Older People) on their territories. To enhance COIC, they took on the mandate of forming strategic partnerships with organisations on their territories such as solo physician clinics, FMGs, network clinics, community organisations and community pharmacies.

## Other PHC reforms

In Quebec, physicians (family physicians and specialists) are independent operators, and have historically been resistant to reforms they perceive as limiting their autonomy and independence [12]. Over the years, physicians have adopted organisational reforms to greater or lesser degrees, depending on their various interests. This section briefly describes two recent reforms aimed at promoting participation of family physicians in the healthcare system. These interventions can potentially enhance the participation of physicians in integrated care and services at the community level by reinforcing their collaborations with the IHSSCs/IUHSSCs.

PHC reforms are highly prioritised in Québec. In a recent Commonwealth Funds survey, 25% of Quebec residents 18 years of age or older reported that they did not have a family physician [16, p.14]. Quebec has the highest rate in Canada of persons reporting not having a family physician [11], hence, most people go directly to the emergency unit of the hospital for their healthcare. To address this problem, government set an objective of ensuring that at least 85% of Quebec’s population was enrolled to a family physician by 31 December 2017. Financial penalties were proposed for family physicians who did not carry their own share, if this target was not met. This law was in line with government policy which aimed to improve COIC by matching every resident of Quebec to a physician. Being enrolled to a PHC provider is considered a key first step for patients to have access to PHC services.

Integrated multidisciplinary practices are the foundation of successful team work and are thought to be an essential component of COIC [17]. To improve the accessibility and quality of COIC in its territory, the government of Québec launched a *funding and professional support program for Family Medicine Groups* [18]. A FMG is defined as a group of family physicians working closely with nurses and other providers in the delivery of care to enrolled patients on a non-geographical basis [19]. This program allocates financial and human resources to eligible FMGs. Human resources include other medical personnel such as nurses, social workers, nutritionists, and occupation therapists, who are employed/paid by the IHSSCs/IUHSSCs to work in local FMGs. The financial and personnel support offered to the FMGs are proportional to the number of patients registered and the types of services offered by the clinic. This is in line with government policy, which aims to improve COIC by promoting grouped medical practices based on interdisciplinary teams, rather than solo practices. It is expected that grouped practices and interdisciplinary teamwork will facilitate patient enrollment, improve the quality of care for local populations, and increase usage of local medical services. This reform encourages FMGs to prioritise vulnerable clients, while linking the IHSSCs/IUHSSCs to FMGs within a continuum of services.

## Discussion

In adopting successive structural reforms, the government of Quebec’s explicit aim was to improve the accessibility, quality, and efficiency of healthcare services [8,10,15]. This paper describes two approaches to improve COIC: (i) the creation of IHSSCs/IUHSSCs through the merger of all public healthcare organisations, and (ii) two strategies to enhance the participation of family physicians in COIC. The IHSSCs/IUHSSCs were mandated by government to create strategic alliances with grouped family medicine clinics of their territories. Two years after the implementation of the 2015 organisational mergers, what lessons have we learnt?

The impact of mergers on healthcare systems have historically had mixed reviews. Some argue that larger organisations are more able to manage joint budgets and achieve economies of scale [20]. Others point out that centralisation of decision-making powers, disempowers grass-roots practices [8,21]. Regarding the 2015 healthcare reforms and extensive mergers in Quebec, it is not clear to what extent senior management of IHSSCs/IUHSSCs can develop or adapt health policies (formerly a prerogative of the Health and Social Services Regional Agencies) to the specific needs of their local populations. How can these new organisations address their local specificities in a context of regional centralised governance? Several authors argue that integration of services must not be achieved through mergers [8,19,22]. Contandriopoulos et al. [8] further point out that contemporary health services research literature suggests that administrative mergers, with the centralisation of decision-making powers that they entail, have not always been proven to improve the accessibility, quality, efficiency or performance of the healthcare system. Merging organisations seems to benefit inter-organisational collaborations at the management/administrative level, but yields fewer benefits at the clinical level, where providers still operate within organisational boundaries [22]. Furthermore, the size of the merged organisation has to be taken into consideration; for instance, there is a body of concurring scientific evidence suggesting that small administrative mergers serving a population of up to 100,000 may be beneficial, while larger organisational mergers may not be very efficient [23]. At this point in time, the IHSSCs/IUHSSCs, which generally provide services to several hundred thousand residents, have far exceeded the threshold of efficiency.

Did the government of Quebec view the centralisation of powers as a powerful lever without which it would not be able to pass through its healthcare system reforms? As previously mentioned, the government modified the duties of family physicians, in a bid to improve access to COIC in Quebec. These reforms were not particularly popular amongst physicians, who wrote a complaint to the health and social services commission [24]. The unprecedented financial penalties and administrative sanctions for family physicians were generally viewed as coercive measures. This led to a backlash from physicians, who vigorously protested what they perceived as a threat to their autonomy [24]. As of February 2018, the penalties have not yet been adopted in Quebec.

The financial and personnel support to FMGs was intended to promote the implementation and sustainability of a ‘grouped practice’ model of COIC at the expense of the solo practice model. This reform also fostered medical and social integration through inter-professional and multidisciplinary collaborations between physicians and other health and social providers such as nurses, social workers, occupational therapists etc. The fact that private FMGs employed other health and social providers through the IHSSCs/IUHSSCs, was expected to improve collaboration between public healthcare and private medical PHC organisations. However, challenges arose in integrating other health and social providers into grouped practice clinics. Their roles and duties in the grouped practices, which are autonomous privately-owned clinics that vary in scope, specialities and missions, were not always properly defined [25–28]. For instance, some FMGs offer general services, others focus on medical specialities, while yet others have a mandate to train residents in medicine. These issues were compounded by the lack of clarity on the hierarchy of personnel in the grouped practices, as acknowledged by the Government of Quebec in their follow-up report [29].

The government has developed monitoring tools and published progress reports on the implementation of COIC initiatives such as the Local Health Network for Older People (LHNOP) [30–32]. For instance, the monitoring tool for the implementation of the LHNOP (*Outil de Suivi de l’Implantation des Réseaux des Services Intégrés aux Personnes Âgées*, OSIRSIPA) [31] presents indicators for the measurement of 9 components of the integrated care model for older people (a joint governing board, case management, a multiclientele assessment tool, an individualised service plan, a health information system, a common access point, a family physician involved in the continuum of care for the older person, an accessible geriatric team, and an administrator responsible for the integrated care organisation). This tool does not address issues such as the funding of the integrated care model, interprofessional collaborations, and patient engagement. At a macro level, there is little evidence-based data to support the sweeping healthcare reforms/mergers of 2015. The government did not provide clear evidence that the 2004 health system reforms/mergers had worked so well that it was necessary to further centralise governance of the province’s health system. In stating ‘there is no credible evidence to suggest that large-scale administrative mergers such as those proposed in Law 10 [the 2015 merger] result in improved accessibility, quality or efficiency [of Quebec’s healthcare system]’ [8, p.14] Contandriopoulos et al. seem to convey the opinion of most observers on the lack of monitoring, evaluation, and evidence supporting government’s pursuit of mergers.

## Conclusion

The successive healthcare system reforms conducted in Quebec are part of an international movement of healthcare system adaptations in response to the demographic changes of an ageing population, economic pressures on the healthcare system, as well as epistemological and conceptual changes in how health is viewed in modern societies. Quebec’s government has chosen to foster COIC through the merger of public healthcare organisations, and the implementation of parallel interventions aimed at promoting the participation of physicians and grouped medical practices. The mechanisms through which centralised healthcare systems achieve COIC need to be properly understood in order to improve meaningful clinical outcomes. Mergers may not sufficiently achieve integration of services in all its dimensions. These reforms should be monitored and evaluated on their capacity to mobilise all health and social care providers, as well as physicians to participate in COIC.

## Governance information

Université de Sherbrooke.

## Disclosure statement

No potential conflict of interest was reported by the authors.

## Funding

Yves Couturier and Mylaine Breton are funded by a Canada Research Chair. This study is supported by grants from the Canadian Institutes of Health Research (Funding Reference Number TTF-128263) and from the New Zealand Health Research Council.
